# Removal of Arsenic (V) from Aqueous Solutions Using Chitosan–Red Scoria and Chitosan–Pumice Blends

**DOI:** 10.3390/ijerph14080895

**Published:** 2017-08-09

**Authors:** Tsegaye Girma Asere, Stein Mincke, Jeriffa De Clercq, Kim Verbeken, Dejene A. Tessema, Fekadu Fufa, Christian V. Stevens, Gijs Du Laing

**Affiliations:** 1 Laboratory of Analytical Chemistry and Applied Ecochemistry, Ghent University, Coupure Links 653, 9000 Ghent, Belgium; Gijs.dulaing@ugent.be; 2 Department of Sustainable Organic Chemistry and Technology, Ghent University, Coupure Links 653, 9000 Ghent, Belgium; stein.mincke@ugent.be (S.M.); Chris.Stevens@ugent.be (C.V.); 3 Department of Chemical Engineering and Technical Chemistry, Ghent University, Valentin Vaerwyckweg 1, 9000 Ghent, Belgium; Jeriffa.declercq@ugent.be; 4 Department of Materials, Textiles and Chemical Engineering, Ghent University, Technologiepark 903, 9052 Zwijnaarde, Belgium; kim.verbeken@ugent.be; 5 Department of Chemistry, Welkite University, Southern Nations, Nationalities and Peoples’ Region, P.O. Box 07, Welkite, Ethiopia; dejeneayele@yahoo.com; 6 Department of Water Resources and Environmental Engineering, Jimma University, P.O. Box 378, Jimma Ethiopia; fekaduff2010@gmail.com

**Keywords:** red scoria, pumice, chitosan, arsenic, adsorption

## Abstract

In different regions across the globe, elevated arsenic contents in the groundwater constitute a major health problem. In this work, a biopolymer chitosan has been blended with volcanic rocks (red scoria and pumice) for arsenic (V) removal. The effect of three blending ratios of chitosan and volcanic rocks (1:2, 1:5 and 1:10) on arsenic removal has been studied. The optimal blending ratio was 1:5 (chitosan: volcanic rocks) with maximum adsorption capacity of 0.72 mg/g and 0.71 mg/g for chitosan: red scoria (Ch–Rs) and chitosan: pumice (Ch–Pu), respectively. The experimental adsorption data fitted well a Langmuir isotherm (*R*^2^ > 0.99) and followed pseudo-second-order kinetics. The high stability of the materials and their high arsenic (V) removal efficiency (~93%) in a wide pH range (4 to 10) are useful for real field applications. Moreover, the blends could be regenerated using 0.05 M NaOH and used for several cycles without losing their original arsenic removal efficiency. The results of the study demonstrate that chitosan-volcanic rock blends should be further explored as a potential sustainable solution for removal of arsenic (V) from water.

## 1. Introduction

Arsenic (As) is released into the aquatic environment through a complex combination of natural biogeochemical reactions and human interactions. Most arsenic related problems are due to mobilization and transport by natural processes such as weathering of rocks and minerals. However, arsenic mobilization can also be caused or aggravated by anthropogenic activities like mining, fossil-fuel combustion and smelting of Cu, Ni, Pb, and Zn ores [1,2,3,4]. Arsenic can occur in the environment in a number of oxidation states (−3, −1, 0, +3 and +5). However, in aqueous systems inorganic arsenic exists mainly in the oxidation states +3 as arsenite and +5 as arsenate, depending on the redox conditions [4]. As (III) is more mobile in natural water and is more toxic than As (V) [5,6]. Exposure to inorganic arsenic is recognized to be a risk for humans because it affects the lungs, skin, liver, kidney, and blood vessels [7,8,9,10]. Thus, it must be removed from drinking water when its concentration is above the safety limit for human consumption (>10 µg/L) [11]. A high concentration of arsenic in drinking water has been reported in countries such as Argentina, Bangladesh, China, Chile, Canada, Hungary, India, Japan, Mexico, Poland, Taiwan, and USA [12,13]. Arsenic concentrations as high as 190 µg/L have also been detected in the aquifers of Ziway and Koka basin, the northern part of the Main Ethiopian Rift Valley (MER) [14]. The field arsenic speciation measurements have revealed that As (V) is much more abundant compared to As (III) in the water of the area [14]. This may be due to the fact that As (III) is easily oxidized to As (V). Therefore, this study was focused on removal of As (V) from water.

Common methods for arsenic removal from water include chemical precipitation, filtration, coagulation, anion exchange, reverse osmosis, adsorption techniques, and use of Donnan membranes [15]. However, the application of membrane techniques, ion exchange, dialysis and chemical treatment techniques require high initial and maintenance costs, and skilled manpower on top of that. Nowadays, adsorption has been recognized as a suitable removal technology mostly for developing regions with the merits of convenient processing, simple operation, potential regeneration, and little toxic sludge generation [7,16].

**Figure 1:**
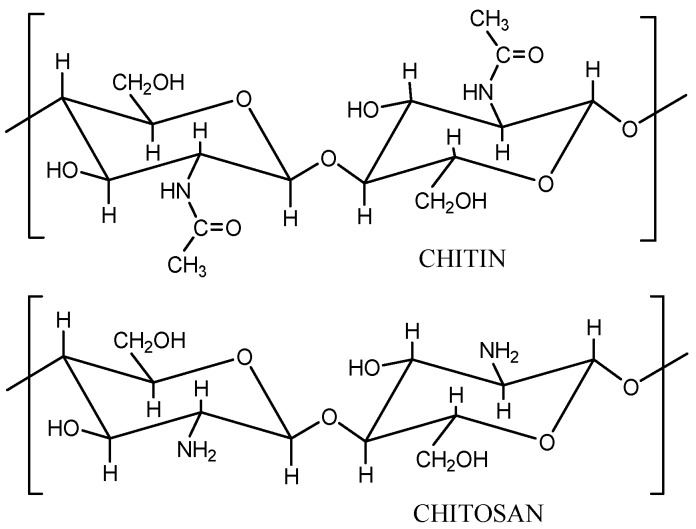
Structure of chitin and chitosan unit [20].

Chitosan and Chitin have been investigated for arsenic removal since the 1980s [17]. Chitosan (poly-β(1-4)-2-amino-2-deoxy-d-glucose) is made by partial deacetylation of chitin [18,19]. Chitin (poly-β(1-4)-2-acetamido-2-deoxy-d-glucose) is an abundant polysaccharide, found in various organisms including fungi, nematodes, and crustacean shells. Commercially, it is extracted from exoskeleton shellfish processing waste. The structural unit for chitin and chitosan is shown in Figure 1.

Recently, these low cost, non-toxic, and biodegradable polymeric materials have received much attention for the treatment of contaminated water and wastewater [18,21] because chitin and chitosan-derivatives possess many amino and hydroxyl groups [18,22]. They have shown good potential for the removal of various pollutants such as arsenic [23], heavy metal ions [24,25], radionuclides [26], dyes [27,28], fluoride [20] and other miscellaneous pollutants from (waste) water. However, chitosan, itself soluble in acidic conditions, has poor mechanical properties for practical applications. Hence, chitosan has been treated with cross-linking agents such as epichlorohydrin [29], tripolyphosphate [30,31], and glutaraldehyde [32,33] or immobilized onto a solid surface to enhance its chemical stability and mechanical strength [34].

Various modifications of chitosan have been made to improve its arsenic adsorption capacity and/or acid stability such as chitosan functionalized with 3,4-diamino benzoic acid [35], chitosan impregnated with metal ions/oxides [36,37,38,39,40,41], zerovalent iron encapsulated in chitosan nanospheres [42], chitosan complexed with different metal ions like Cu (II), Fe (III), La (III), Mo (VI) and Zr (IV) [43], chitosan coated on ceramic alumina [7], and chitosan immobilized onto sodium silicate [44]. Therefore, the main objective of the present study was to prepare chitosan-red scoria (Ch–Rs) and chitosan-pumice (Ch–Pu) blends and evaluate their performance for As (V) removal. The efficiency of Ch–Rs and Ch–Pu blends was compared with that of red scoria, pumice, chitosan powder and chitosan gel, separately. Moreover, the effect of interfering ions was studied.

## 2. Materials and Methods

### 2.1. Adsorbent Preparation

Pumice and scoria, volcanic rocks, are abundant in many parts of the world. Pumice is a light porous igneous volcanic rock with large surface area and high water adsorption capacity (20–30%), whereas scoria is formed of vesicular fine to coarse fragments, reddish or black color, and light size [45,46]. The rock samples were collected from volcanic cones of the Main Ethiopian Rift Valley (MER), Ethiopia; approximately 100 km East of Addis Ababa. The collected adsorbent samples were washed several times with deionized water to remove any impurities and subsequently dried at 55 °C for 48 h [47]. The dried adsorbents were crushed in a mortar and sieved to obtain a silt fraction (<0.075 mm) [45,46].

The chitosan blend was prepared, with slight modification, according to the method used by Turan et al. [34]. Chitosan powder (low molecular weight, Sigma-Aldrich, St. Louis, MO, USA) was dissolved in 2.0% (*v*/*v*) acetic acid in order to obtain a 4% (m/v) chitosan solution. The mixture was stirred until a clear solution was obtained. About 60.0 g of red scoria silt particle size (<0.075 mm) was added slowly to the chitosan solution and stirred for 2 h. Then, 0.1 M NaOH was added to neutralize the excess acetic acid and the blend was washed several times with deionized water. The blend was soaked in aqueous hydrochloric acid (pH ≈ 4.5) in order to protonate the amine function of chitosan. After rinsing with deionized water, the blend was dried at 70 °C for 48 h and ground by a mortar and pestle prior to use. The same procedure was used to prepare a chitosan–pumice blend. Chitosan (without volcanic rocks) was prepared following the above procedure and called chitosan gel (dry).

### 2.2. Adsorbent Characterization

#### 2.2.1. Chemical Composition

The elemental composition of the geomaterials was analyzed using inductively coupled plasma-optical emission spectroscopy (ICP-OES, Varian Vista MPX, Palo Alto, CA, USA). Energy dispersive X-ray (EDX) spectroscopy (Silicon Drift Detector (SDD) X-MaxN, Oxford Instruments, Abingdon, Oxfordshire, UK) was employed to obtain information on the oxide contents of the geomaterials. The surface morphology was studied by scanning electron microscope (FEG SEM JSM-7600F, JEOL, Peabody, MA, USA). Pyrolysis of the blends was carried out using a muffle furnace to determine the chitosan loading on red scoria and pumice [48]. The Brunauer-Emmett-Teller (BET) specific surface area of adsorbents was determined from N_2_ gas adsorption/desorption isotherms obtained using BEL, Japan, Inc. Belsorp mini-II. The leaching of the chitosan from the blends was analyzed using a total organic carbon (TOC) analyzer (Shimadzu TOC-VCPN, Columbia, MD, USA).

#### 2.2.2. Determination of pH and Point of Zero Charge

The pH of the adsorbents was measured using a pH meter in a 1:10 adsorbent/water ratio according to the standard method [49,50]. The point of zero charge (pH_pzc_) of adsorbents was determined using a 0.01 M and 0.1 M solutions of NaCl as an electrolyte and adding 0.1 M solutions of NaOH or HCl [51,52].

### 2.3. Chemicals

A 1000 mg/L As(V) stock solution was prepared by dissolving an appropriate amount of Na_2_HAsO_4_·7H_2_O (Merck KGaA, Darmstadt, Germany) in deionized water. Synthetic arsenic containing water used for the adsorption experiments was prepared by diluting the stock solution with deionized water to obtain an arsenic concentration in the range of 0.1 mg/L–25 mg/L. Solutions of bicarbonate, chloride, nitrate, sulfate and phosphate anions were prepared from their respective sodium salts.

### 2.4. Analytical Procedures

Inductively coupled plasma-mass spectrometry (ICP-MS, ELAN DRC-e, Perkin Elmer SCIEX, Waltham, MA, USA) instrument was used in this study. The ICP and DRC (dynamic reaction cell) conditions were selected that maximized the ion signals of arsenic while reducing the background to a minimum. To remove possible interferences, the arsenic content was determined as AsO after reaction of As with oxygen using ICP-MS in DRC mode. The operating parameters of the ICP-MS used for this work are: Rf power 1300 W, plasma gas flow 15 L/min, auxiliary gas flow 1.2 L/min, nebulizer gas flow 0.7 L/min, O_2_ gas flow 0.4 mL/min, and 1 reading in 3 replicates. ICP-MS ELAN software (Version 3.4, Perkin Elmer SCIEX, Waltham, MA 02451, USA) was used to control the instrument and process the acquired data. A 100 mg/L stock solution of ICP Multi-element standard solution XVI was obtained from Merck, Germany. Milli-Q water (18.2 MΩ cm^−1^) was used for the preparation of all blanks, standards and sample solutions. Blanks, standards and sample solutions were diluted 10 times with a solution containing 10 μg/L internal standard (Ga and Rh). The signal of the internal standards was used to monitor the consistency of the measurements. Linear calibration curves with a minimum regression coefficient *R*^2^ of 0.9999 were obtained using standards in the range of 0.05 μg/L up to 20 μg/L As. For quality assurance, recalibrations were done after every 15–20 samples. The degree of the reproducibility was also assured by neglecting data with relative standard deviation (RSD) >10%. The limits of detection and quantification for arsenic were 0.11 µg/L and 0.30 µg/L, respectively.

### 2.5. Batch Arsenic Adsorption Studies

The batch adsorption experiments were performed in triplicate using 50 mL polyethylene plastic centrifuge tubes. About 0.200 g of dry powdered sorbent (Ch–Rs or Ch–Pu) was added to 25 mL of known As (V) concentration having a desired pH value. The initial pH was adjusted using HCl and/or NaOH. Then, the tubes were agitated at 200 rpm on a horizontal shaker at 25 °C for the desired period of time. Aqueous samples were taken after predetermined time intervals and were filtered through a 0.45 μm membrane filter to separate the adsorbent from the As (V) containing solution. Then, the residual arsenic concentration in the filtrate was measured using ICP-MS.

The As (V) adsorption capacity, *q_t_* (mg/g) at time *t* (min), and the As (V) removal efficiency (% adsorption) were determined using the Equations (1) and (2) respectively.

(1) $$q_{t}=\left(C_{0}-C_{t}\right)\left(\frac{V}{W}\right)$$

(2) $$Adsorption\left(\%\right)=\left(\frac{C_{0}-C_{t}}{C_{0}}\right)\times 10$$

where *C_0_* and *C_t_* (mg/L) are the initial arsenic concentration and the concentration at time *t* (min), respectively, *V* is the solution volume (L) and *W* (g) is the adsorbent mass.

### 2.6. Regeneration of the Spent Adsorbents

First, saturated adsorbent is produced by adsorbing 0.25 mg/L As (V) solution. Then, the loaded adsorbents were filtered and dried at 70 °C for 24 h. Next, 25 mL of a 0.05 M NaOH solution was added to the centrifuge tube and the tubes were shaken at 200 rpm on a horizontal shaker at 25 °C for 2 h. Subsequently, the suspensions were filtered and the arsenic content of the filtrate was measured. The adsorbent was soaked in aqueous HCl at pH ≈ 4.5, rinsed and dried at 70 °C for 24 h for re-use.

## 3. Results and Discussion

### 3.1. Adsorbent Characterization

#### 3.1.1. Chitosan Loaded on Rs and Pu

Pure red scoria and pumice lost about 0.15 and 8.35 wt % during pyrolysis, respectively. Pure chitosan leaves a residue of about 0.09 wt % after pyrolysis at 750 °C. Blends of chitosan with red scoria and pumice (1:5 ratios) lost 13.26 wt % and 21.70 wt %, respectively. Accordingly, the net weight loss for Ch–Rs was 13.02% and Ch–Pu was 13.26%. These weight losses should correspond to the amount of chitosan in the final blends. They are only about 3% lower than the initial amount of chitosan used to prepare the blends (~16%), which illustrates that the method used for coating the red scoria and pumice with chitosan is effective.

#### 3.1.2. Chemical Composition

Si, Al and Fe are the major elements in pumice and red scoria (Table 1) as determined by ICP-OES and confirmed by EDX. Other elements, except K (3.9%) in pumice and Ca (6.3%) in red scoria, were present in relatively smaller amounts or below the detection limit of the instrument. The EDX measurement indicated that the oxides of Si, Fe, and Al were the major constituents of both red scoria and pumice. Similar values were reported by Alemayehu and Lennartz [46].

**Figure 2:**
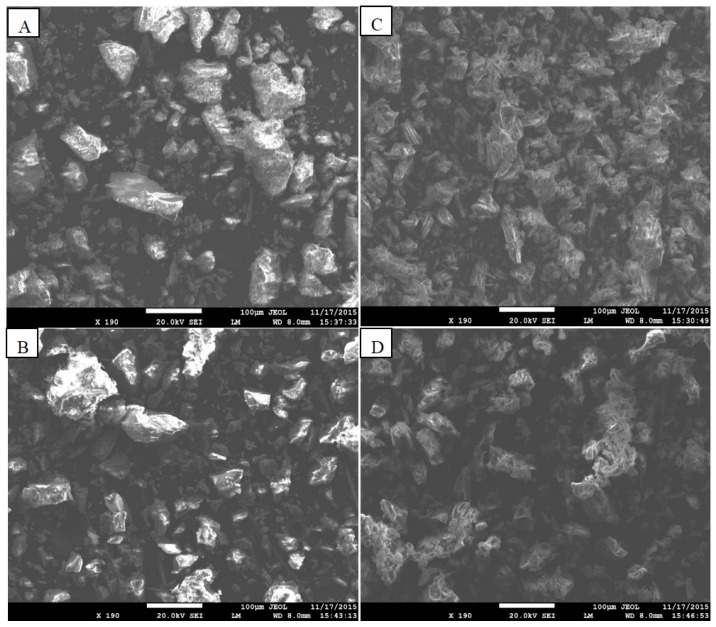
SEM images of (**A**) red scoria, (**B**) Ch–Rs, (**C**) pumice, and (**D**) Ch–Pu.

SEM images of red scoria, Ch–Rs, pumice and Ch–Pu are shown in Figure 2. No differences in morphology are observed between chitosan-coated (1:5 ratio) and uncoated materials, which illustrates that isolated chitosan particles were not formed during the blending processes. This may further indicate that the chitosan is well dispersed on the surface of the red scoria and pumice. The specific surface areas of the red scoria and pumice and Ch–Rs and Ch–Pu blends were found to be 2.63, 3.88, 1.01, and 1.5 m^2^/g, respectively. The observed decrease in the surface area of the blends as compared to the natural materials may be caused by the chitosan blocking some of the naturally available pores in red scoria and pumice.

#### 3.1.3. pH and Point of Zero Charge

The pH of the adsorbents measured in water was found to be 7.8 (red scoria), 9.4 (pumice), 5.3 (Ch–Rs), and 5.8 (Ch–Pu), respectively. The high equilibrium pH values of both red scoria and pumice imply that non-modified red scoria and pumice may not be favorable adsorbents for anions such as arsenate. The pH_pzc_ for red scoria and pumice was 7.5 and 6.8, respectively, whereas the pH_pzc_ of Ch–Rs and Ch–Pu (1:5 ratio) was found to be 6.5 and 6.6, respectively. Similar values of pH_pzc_ were reported for pumice by Sepehr et al. [53] and for protonated chitosan by Padilla-Rodriguez et al. [52]. In this work, the pH in water as well as the pH_pzc_ of chitosan blends were found to be lower than that of unmodified red scoria and pumice.

### 3.2. Adsorption of As (V) on Ch–Rs and Ch–Pu Blends

#### 3.2.1. Preliminary Adsorption Experiments

**Figure 3:**
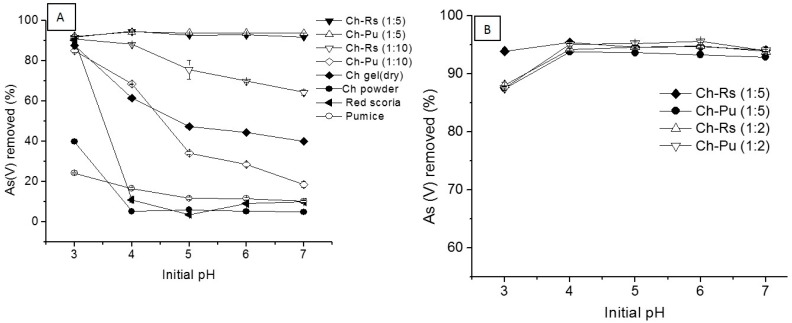
Removal of As (V) by different adsorbents: (**A**) As(V) concentration of 1.0 mg/L, 20 g/L adsorbent dose, shaking for 24 h, at 24 ± 1 °C, (**B**) As(V) concentration of 0.25 mg/L, 8 g/L adsorbent dose, shaking for 2 h, at 24 ± 1 °C.

Preliminary adsorption experiments were carried out under identical experimental conditions using chitosan powder, chitosan gel (dry), pumice, red scoria, Ch–Rs and Ch–Pu blends at three different Ch/Rs and Ch/Pu ratios (1:2, 1:5, and 1:10). The percentage of As (V) removed using these adsorbents is shown in Figure 3A,B. A protonated chitosan gel exhibited enhanced As (V) adsorption compared to chitosan powder (maximum ~88% versus ~40% at pH 3). Padilla-Rodriguez et al. [54] also found that protonated chitosan flakes were more effective than chitosan powder for the removal of As (V) from aqueous solutions. Both the Ch–Rs and Ch–Pu blends in 1:2 and 1:5 ratios showed the highest As(V) removal efficiency (>90%) at a wide pH range (3 to 7) as compared to the unmodified red scoria (~90% only at pH 3) and pumice (<20%), as well as the blends at 1:10 ratio of chitosan–red scoria (>90% at pH 3 up to ~65% at pH 7) and chitosan–pumice (~85% at pH 3 up to ~19% at pH 7). The lower removal efficiency of red scoria and pumice at initial pH values between 4 and 7 can be associated to the relatively high pH reached at equilibrium (8.9–9.3) during arsenate adsorption. Since the equilibrium pH is greater than the pH_pzc_ of red scoria and pumice (7.5 and 6.8, respectively), their surface is negatively charged at equilibrium and could not bind arsenate efficiently. Similar findings were reported by Turan et al. [34] as chitosan-immobilized pumice exhibited >90% of As (V) removal at initial pH 3.0–7.0 whereas pumice displayed <20% adsorption toward As (V) and chitosan removed ~90% of As (V) only at initial pH 3.0. The results demonstrate the advantage of immobilizing chitosan onto solid supports such as pumice and red scoria. Hence, further experiments for optimization of various adsorption parameters were carried out using the Ch–Rs (1:5) and Ch–Pu (1:5) blends.

#### 3.2.2. Effect of Contact Time

**Figure 4:**
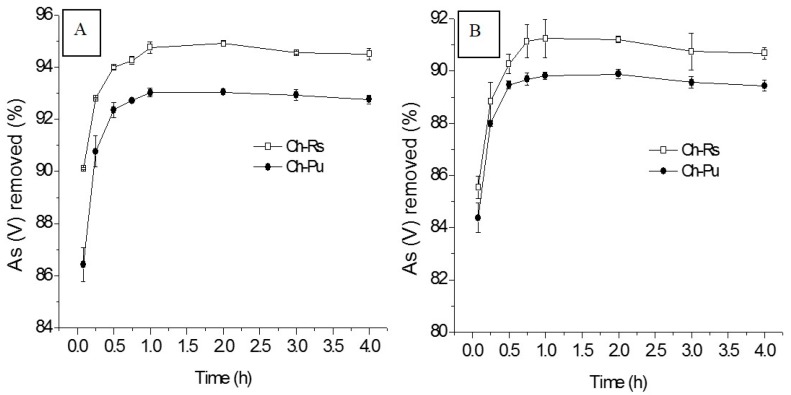
Effect of contact time on removal of As(V) by Ch–Rs and Ch–Pu blends at initial As (V) concentration of (**A**) 0.25 mg/L and (**B**) 2.0 mg/L (adsorbent dose 8 g/L, initial pH 7, shaking at 200 rpm at 24 ± 1 °C).

Adsorption of As (V) by Ch–Rs and Ch–Pu is very rapid, with more than 85% of the initial amount of arsenic being removed within 5 min (Figure 4A,B). This rapid adsorption in the beginning can be attributed to the greater concentration gradient and the availability of a lot of adsorption sites. This is a common behavior in adsorption processes and has been reported in other studies [37,55]. After 30 min, further changes of the adsorption capacity were negligible. However, to ensure maximum adsorption at equilibrium, a contact time of 2 h was chosen for subsequent optimization of other adsorption parameters. At equilibrium, the As (V) adsorption capacities of the Ch–Rs and Ch–Pu blends were 0.030 mg/g (94.9%) and 0.028 mg/g (93.1%), respectively.

#### 3.2.3. Adsorption Kinetics

**Figure 5:**
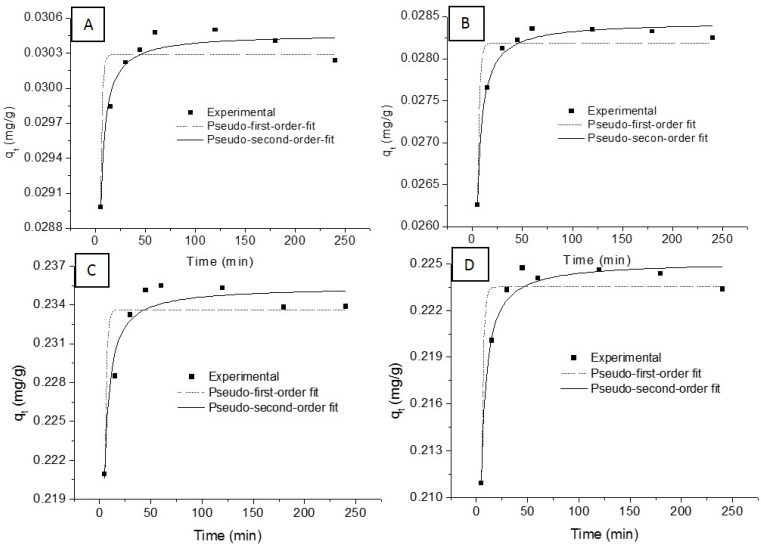
Pseudo-first-order and pseudo-second-order kinetic model fits and experimental kinetic data: (**A**) Ch–Rs (0.25 mg/L As(V)), (**B**) Ch–Pu (0.25 mg/L As(V)), (**C**) Ch–Rs (2.0 mg/L As(V)), and (**D**) Ch–Pu (2.0 mg/L As(V)) (initial pH 7, 8 g/L adsorbent, shaking at 200 rpm at 24 ± 1 °C).

In order to investigate the adsorption rates of As (V) on the Ch–Rs and Ch–Pu adsorbents, the adsorption process was determined by using pseudo-first order and a pseudo-second order kinetic models. The nonlinear expressions of the pseudo-first-order and pseudo-second-order [56] models are given in Equations (3) and (4), respectively.

(3) $$q_{t}=q_{e\;}\left(1-\;exp^{-K_{1}t}\right)$$

(4) $$q_{t}=\frac{k_{2}q_{e}^{2}t}{1\;+\;k_{2}q_{e}t}$$

where, *k*_1_ (min^−1^) is pseudo-first-order rate constant, *k*_2_ (g mg^−1^ min^−1^) is pseudo-second-order rate constant, *q_t_* and *q_e_* are the arsenate adsorption capacity (mg/g) at any time *t* (min) and at equilibrium, respectively. The pseudo-first-order and pseudo-second-order arsenic adsorption kinetics fit is shown in Figure 5A,B, and the values of *k*_1_, *k*_2_, *q_e,cal_* (calculated), and *q_e,exp_* (experimental) are presented in Table 2.

The analysis of the kinetics data showed that the values of *q_e,cal_* and *q_e,exp_* were very similar for Ch–Rs and Ch–Pu. It is clear from Table 2 that the kinetic data could be well explained by a pseudo-second-order model compared to pseudo-first-order model as it has a high correlation coefficient and low *χ*^2^ values for both kinds of adsorbents. Therefore, it can be concluded that the adsorption of As (V) on Ch–Rs and Ch–Pu blends follows pseudo-second-order kinetics.

Another important parameter that provides information about the adsorption rate, particularly at the beginning of the adsorption process, is the adsorption affinity (*V*_0_ = *k*_2_*q_e_*^2^) [57]. The adsorption affinity (*V*_0_) increases as the initial As (V) increases. The similar values of *V*_0_ for Ch–Rs and Ch–Pu (Table 2) indicate comparable adsorption affinity of Ch–Pu and Ch–Rs blends. However, this cannot explain the rate-limiting step. The rate-limiting step may either be the boundary layer (film) or the intraparticle (pore) diffusion of the solute from the bulk of the solution in a batch process [58]. Therefore, the diffusion mechanism of the As (V) molecules was also examined by applying the Weber and Morris intraparticle diffusion model [59] that is given by (Equation (5)).

(5) $$q_{t}=k_{p}t^{0.5}\;+\;c$$

**Figure 6:**
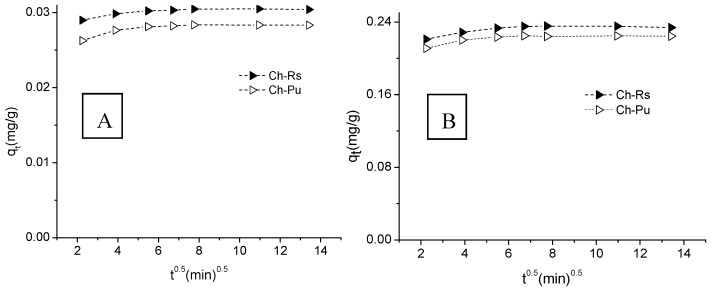
Intraparticle diffusion plots of As(V) adsorption on Ch–Rs and Ch–Pu blends at initial As(V) concentration of (**A**) 0.25 mg/L and (**B**) 2.0 mg/L.

*k*_p_ (mg/(g min^0.5^)) is the intraparticle diffusion rate constant; and *c* (mg/g) is the intercept of the intraparticle diffusion model. If the rate-limiting step is the intraparticle diffusion, the plot of *q*_t_ versus *t*^1/2^ should be a straight line and pass through the origin [58]. The curves of *q_t_* versus *t*^0.5^ are given in Figure 6A,B. It is clear that each curve shows a multi-linear plot. Moreover, each curve does not pass through the origin, which indicates that more than one mechanism/process (and not solely intraparticle diffusion) is determining the rate of the removal process [60].

#### 3.2.4. Influence of pH

**Figure 7:**
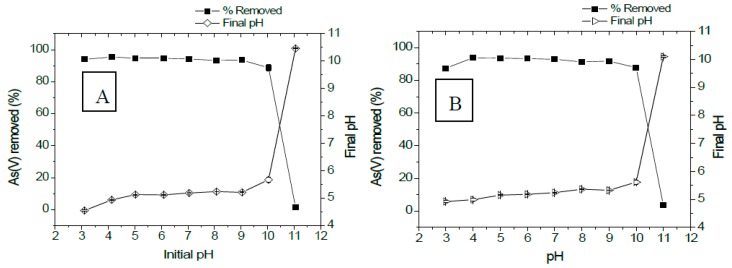
Effect of initial pH on removal of As(V) by (**A**) Ch–Rs (**B**) Ch–Pu blends (initial As(V) concentration 0.25 mg/L, adsorbent dose 8 g/L, shaking 2 h at 200 rpm at 24 ± 1 °C).

**Scheme 1:**
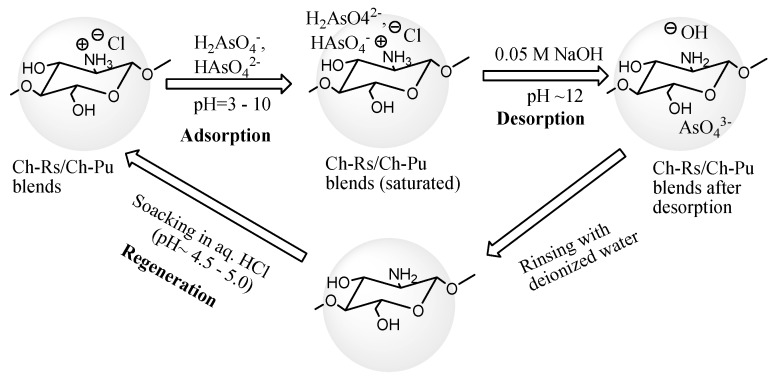
Possible mechanism of adsorption and desorption of arsenate ions on Ch–Rs and Ch–Pu blends.

The pH of the solution can significantly affect the sorption of anions on the adsorbents by changing the degree of ionization, the speciation of the ions and the surface charge of the adsorbent. Therefore, adsorption of As (V) was studied at nine different initial pH values, between 3 and 11, while keeping the other parameters constant (Figure 7A,B). The initial solution pH was adjusted by adding a 0.1 M HCl and/or NaOH. Both Ch–Rs and Ch–Pu have a high adsorption efficiency (89–95%, Ch–Rs and 88–94%, Ch–Pu) in a wide initial pH range between 3 and 10. Similar results were previously reported by Turan et al. [34] for chitosan-immobilized pumice, >90% of As (V) removed at initial pH 3.0–7.0 and >70% of As (V) adsorption at pH > 8.0. Figure 7A,B also indicate that pH ≥ 11 is suitable for desorption of As (V) saturated Ch–Rs and Ch–Pu blends. Figure 7 shows that the final pH was in the range of 4.6–5.7 (Ch–Rs) and 4.9–5.6 (Ch–Pu) for an initial pH of 3 to 10, while the values of pH_pzc_ were 6.5 and 6.6 for Ch–Rs and Ch–Pu, respectively. Since the final pH is below the pH_pzc_ of the sorbents, the surface of both sorbents will be positively charged throughout the sorption process when the initial pH is below 10 (pH < 10). This indicates that the adsorption mechanism is an electrostatic interaction [22] between the protonated amine group of chitosan (–NH_3_^+^) on the surface of blends and the negatively charged arsenate anions (Scheme 1).

The TOC of treated water showed that the highest solubility of chitosan in the blends was at pH 3 where 7.59% of Ch–Pu and 4.60% of Ch–Rs were found to be soluble. The solubility of chitosan in the Ch–Rs blend varied from 0.94% to 0.96% between pH 4 and 10, whereas the solubility of chitosan in the Ch–Pu blend further decreased from 0.27% at pH ≈ 4.0 to 0.18% at pH ≈ 10.0. The removal capacities of both adsorbents were not significantly affected by the initial pH in the pH range between 3 and 10. Consequently, pH 7 ± 0.1 was chosen for optimization of the other adsorption parameters.

#### 3.2.5. Optimization of Adsorbent Dose

**Figure 8:**
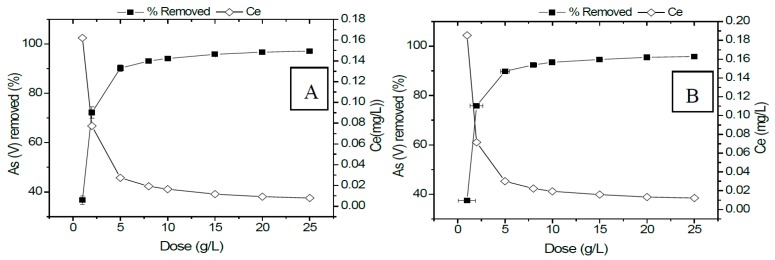
Effect of adsorbent dose on removal of As(V) by (**A**) Ch–Rs (**B**) Ch–Pu blends (initial As(V) concentration 0.25 mg/L, pH 7, shaking 2 h at 200 rpm at 24 ± 1 °C).

The effect of the adsorbent dose on the As (V) removal was studied using different amounts of adsorbent (1–25 g/L) and a fixed As (V) concentration of 0.25 mg/L at pH ≈ 7.0. The As (V) removal efficiency increased rapidly as the dose increased from 1 g/L to 5 g/L, and marginally thereafter (Figure 8A,B). The increase in adsorption efficiency with an increase of the adsorbent dose is due to the greater availability of active surface sites for arsenate binding at a higher adsorbent dose [61]. However, the increase of the removal efficiency was found to be negligible above an adsorbent dose of 8 g/L, which may be considered as the optimum dose. A dose of 20 g/L or higher can lower the arsenate concentration of 0.25 mg/L to a concentration below the WHO guideline for drinking water of 0.01 mg/L. On the other hand, the As (V) adsorption capacity (*q_e_*) decreased from 0.11 to 0.01 mg/g with increasing adsorbent dose for the fixed arsenic concentration (Figure 8A,B). The decrease in the adsorption capacity is due to the increased solid dose for the fixed solute load resulting in a lower availability of arsenate ions per unit mass of adsorbents [61]. This finding is essential for real application of the blends for treating arsenic contaminated drinking water, particularly, in developing countries since pumice, red scoria, and chitosan are low-cost and naturally abundant materials.

#### 3.2.6. Effect of Initial Concentration

**Figure 9:**
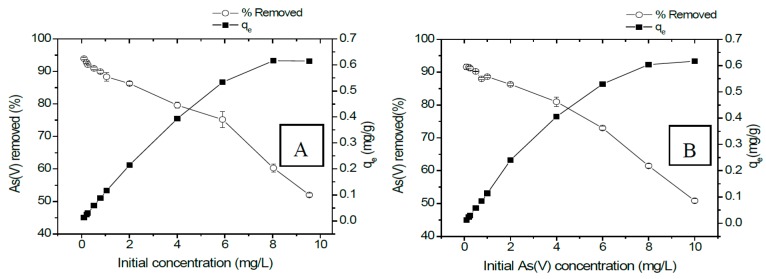
As (V) removed (%) and adsorption capacity equilibrium (mg/g) as function of initial concentration of As(V): (**A**) Ch–Rs (**B**) Ch–Pu blends (adsorbent dose 8 g/L, initial pH 7, shaking 2 h at 200 rpm at 24 ± 1 °C).

The effect of the initial concentration on As (V) removal was investigated by testing various initial sorbate concentrations (0.1 mg/L to 10 mg/L). The As (V) removal percentage decreased with increasing initial arsenic concentration (Figure 9A,B). The decrease in adsorption is attributed to the higher ratio of arsenic ions over available active surface sites with increasing initial arsenic concentration at constant mass of the adsorbent. However, the adsorption capacity increased with increasing initial As (V) concentration until the maximum adsorption capacity was reached (Figure 9A,B). This is commonly observed phenomenon in adsorption processes and has also been reported in other studies [61].

#### 3.2.7. Adsorption Isotherm

The equilibrium experimental data of arsenic adsorption on Ch–Rs and Ch–Pu were fitted to two common isotherm models: Langmuir and Freundlich isotherms that are given in Equations (6) and (7), respectively.

(6) $$q_{e}=\frac{Q_{max}bC_{e}}{\left(1+bC_{e}\right)}$$

(7) qe=KFCe1n

where *C_e_* (mg/L) is the As (V) concentration in the aqueous phase at equilibrium; *q_e_* (mg/g) is the As (V) adsorption capacity at equilibrium; *Q_max_* (mg/g) is the maximum adsorption capacity based on the Langmuir equation; *b* (L/mg) is the Langmuir constant; *K_F_* (mg^1−1/*n*^ L^1/*n*^/g) is the adsorption coefficient; ^1^/*n* is the adsorption intensity.

**Figure 10:**
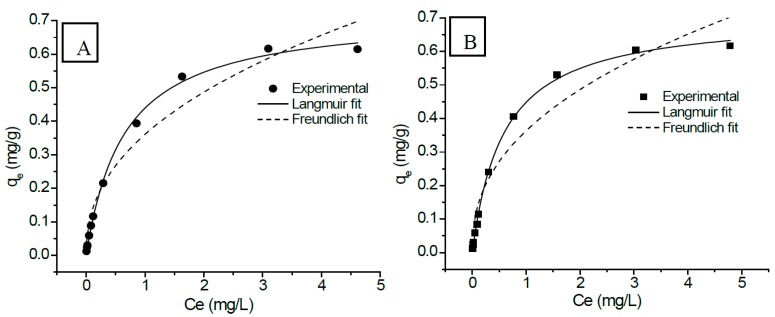
Isotherms of equilibrium adsorption of As (V) on (**A**) Ch–Rs and (**B**) Ch–Pu blends.

Besides the coefficient of determination, the nonlinear chi-squared (*χ*^2^) statistic test was used to identify the best fit model to the observed experimental equilibrium isotherm data. *χ*^2^ is computed using Equation (8) [62].

(8) $$\chi^{2}=\sum {\left(q_{e}-q_{e,cal}\right)}^{2}/q_{e,cal}$$

where *q_e,cal_* (mg/g) is the equilibrium capacity calculated from the model; and *q_e_* (mg/g) is the experimental equilibrium capacity. A small *χ*^2^ value indicates similarity between the modeled and the experimental data, whereas a larger *χ*^2^ value implies variation between the modeled and experimental data [62]. The isotherm plots are graphically presented in Figure 10A,B. The values of the equilibrium constants, *χ*^2^ and *R*^2^ for each isotherm model are presented in Table 3.

The Langmuir isotherm led to a higher correlation coefficient, *R*^2^ > 0.99, and lower chi-square values for Ch–Rs and Ch–Pu blends, *χ*^2^ ≈ 8.34 × 10^−3^ and 8.24 × 10^−3^, respectively. The Langmuir isotherm equation resulted in a maximum adsorption capacity of 0.72 mg/g and 0.71 mg/g, for Ch–Rs and Ch–Pu, respectively. The maximum sorption capacities of Ch–Rs and Ch–Pu adsorbents calculated from the Langmuir isotherm for As (V) in this study are within the same range of both natural and modified adsorbents reported in previous work (Table 4).The fitting of the equilibrium data to the Langmuir isotherm was indicative for a monolayer adsorption of As (V) on the homogeneous surface sites of both Ch–Rs and Ch–Pu blends. The separation factor (*R_L_*) of the Langmuir isotherm can be computed by *R_L_* = 1/(1 + *bC*_0_) [58], where *C*_0_ (mg/L) is the initial As(V) concentration; and b (L/mg) is the Langmuir constant. The adsorption process is irreversible if *R_L_* = 0, favorable if 0 < *R_L_* < 1, linear if *R_L_* = 1 and unfavorable if *R_L_* > 1. The obtained values of *R_L_* are within the range 0–1 (Table 3), indicating favorable equilibrium adsorption of As (V) on both Ch–Rs and Ch–Pu blends [58].

#### 3.2.8. Effect of Co-Existing Anions

**Figure 11:**
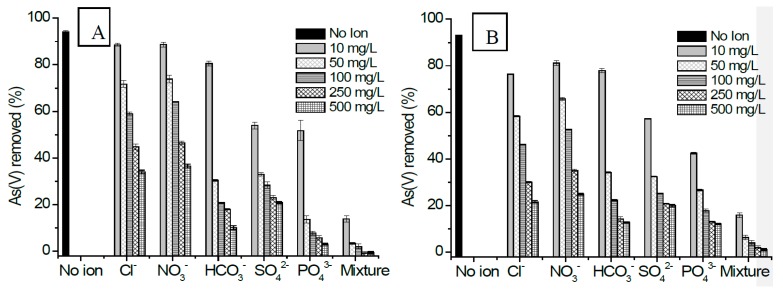
Effect of co-existing anions on As(V) removal by (**A**) Ch–Rs (**B**) Ch–Pu blends (initial concentration 0.25 mg/L, dose 8 g/L, shaking at 200 rpm for 2 h, at 24 ± 1 °C).

The data reported in the previous sections have been gathered with aquatic solutions only containing arsenate ions. This should allow to compare the data, e.g. maximum adsorption capacity, with literature data collected under similar conditions. However, arsenic contaminated water typically contains also several other anions that can affect the adsorption process and compete with the arsenate ions for adsorption. In order to understand the effect of interfering ions, additional adsorption experiments were carried out in presence of 10 mg/L, 50 mg/L, 100 mg/L, 250 mg/L and 500 mg/L salt solutions of chloride (Cl^−^), nitrate (NO_3_^−^), bicarbonate (HCO_3_^−^), sulphate (SO_4_^2−^) and phosphate (PO_4_^3−^), separately and in a mixture [78]. Cl^−^, SO_4_^2−^, NO_3_^−^, HCO_3_^−^ and PO_4_^3−^ ions, separately as well as the mixture of these ions, showed a negative effect on the removal of arsenic (Figure 11A,B). The variations in adsorption capacity of both Ch–Rs and Ch–Pu with varying background electrolyte concentrations indicate that arsenate is predominantly removed by an outer-sphere complexation mechanism. This effect is greater when all six anions are present, revealing that all ions are competing for the active sites. Since the equilibrium pH is below the pH_PZC_, electrostatic interaction is expected to be the dominant removal mechanism. Moreover, as the concentration of the co-existing ions increased from 10 mg/L to 500 mg/L, the removal of arsenic dropped sharply. This is caused by the higher competing effect of these co-existing anions for the active sites of the adsorbents. In general, the percentage of As (V) removal also decreased with an increase in charge of the interfering ion. Therefore, PO_4_^3−^ was the anion that caused the greatest reduction in As (V) adsorption, as was also reported in other studies for various adsorbents [58,61]. However, phosphate is absent or usually present at lower concentration (<0.21 mg/L) in groundwater of Ethiopia [50]. Because of the high bicarbonate content of the Ethiopian Rift Valley groundwater wells (162–2045 mg/L) and lakes water (267–30,376 mg/L) in the Ziway-Shala basin (arsenic—affected areas) [14], bicarbonate could be considered as a potential interfering ion in removal of As(V) from real water samples of those areas using Ch–Rs and Ch–Pu. Taking into account the impact of competing ions on the adsorption process, it may be useful to conduct future adsorption studies with the Ch–Rs and Ch–Pu blends using solutions containing background salts at realistic concentrations and real water samples of arsenic-affected areas.

#### 3.2.9. Desorption of As (V) from Ch–Rs and Ch–Pu Surfaces

**Figure 12:**
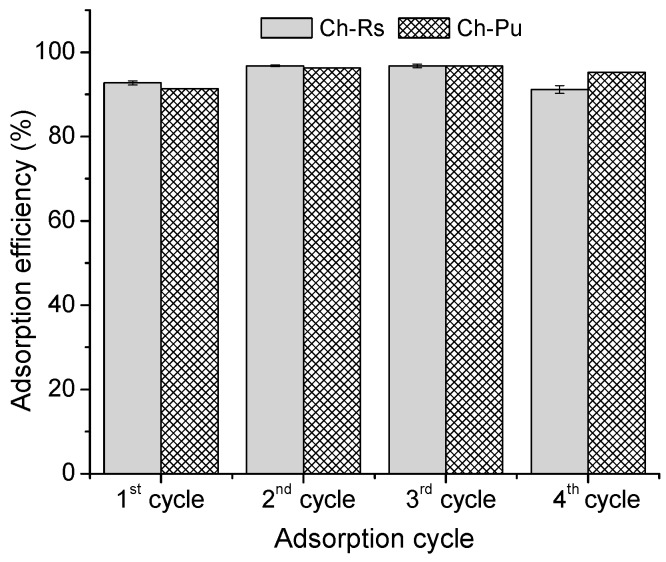
As(V) adsorption cycles of Ch–Rs and Ch–Pu blends.

The possibility to regenerate adsorbents is a key asset when developing a cost-effective adsorbent for pollutant removal from aqueous environments. In the present study, desorption tests were conducted using 0.05 M NaOH solution. The percentages of As (V) that could be desorbed were 92.5–99.4% and 95.7–99.6% for Ch–Rs and Ch–Pu, respectively. Regeneration was studied in 4 adsorption–desorption cycles. Even in the 4th cycle, 98% (Ch–Rs) and ~100% (Ch–Pu) of the actual adsorption efficiency for As (V) was retained (Figure 12). Wang et al. [38] demonstrated that magnetic nanoparticles impregnated chitosan beads could retain about 88.2% of the original As (V) adsorption capacity after 5 cycles of reuse. Another previous study also concluded that a chitosan bed could be recycled up to 15 cycles without losing its initial efficiency [79]. In basic solutions (see Figure 7), the electrostatic interaction between chitosan and the arsenate ions becomes much weaker due to the neutralization of positively charged amino groups (Figure 7A,B). As a consequence, the adsorbed arsenate ion leaves the adsorption site of chitosan (Scheme 1). Subsequently, the adsorbents is regenerated when being in contact with acid through protonation of the amine functional group present in chitosan. This process does not seem to destroy the chitosan skeleton itself, which is a major asset indicating the potential of Ch–Pu and Ch–Rs adsorbents for sustainable treatment of As (V) contaminated water.

## 4. Conclusions

The effectiveness of blending chitosan with red scoria and pumice was confirmed by the small (~3%) loss of chitosan in the blending process. It was observed that blending ratio between the chitosan and the volcanic rocks has a significant influence on arsenic removal efficiency. The optimal blending ratio was 1:5 (chitosan: volcanic rocks) with a maximum adsorption capacity of 0.72 mg/g and 0.71 mg/g for Ch–Rs and Ch–Pu, respectively. Both Ch–Rs and Ch–Pu blends can remove about 93% of 0.25 mg/L As (V) solution in a wide range of initial pH values, avoiding the need for pH adjustment in real applications. A dose of 20 g/L or higher of Ch–Rs/Ch–Pu can lower arsenic concentrations from 0.25 mg/L As (V) to below the WHO guideline (0.01 mg/L) within 2 h. The adsorption was very fast, reaching equilibrium within 30 min, and followed pseudo-second-order kinetics. The similar adsorption performance of the Ch–Rs and Ch–Pu blends suggested that the adsorption occurs on the chitosan surface of the blends, whereas the volcanic rocks mainly serve as supporting material. The experimental equilibrium adsorption data of both Ch–Rs and Ch–Pu fit well to the Langmuir model, which is also an indication for adsorption on a homogeneous surface of protonated chitosan. The high As(V) removal performance of the prepared chitosan blends during several adsorption–desorption cycles, without losing their original capacity, together with the high desorption efficiency (93–99%) also indicate that the adsorbents could be considered as a sustainable solution for the removal of As(V) from drinking water. However, taking into account the impact of competing ions on the adsorption process, it would be useful to conduct future adsorption studies in batch and column modes using solutions containing background salts at realistic concentrations and real water samples of arsenic-affected areas. This should allow to further evaluate the potential of the Ch–Rs and Ch–Pu blends as As (V) adsorbents.

## Figures and Tables

**Table 1:** Elemental composition and oxide content of red scoria and pumice.

Elements	Pumice % (wt)	Red Scoria % (wt)	Oxides	Pumice % (wt)	Red Scoria % (wt)
Si	27.3	18.4	SiO_2_	69.2	42.2
Al	5.6	9.7	Al_2_O_3_	11.9	18.4
Fe	3.3	8.1	FeO	5.8	13.0
K	3.9	0.3	CaO	0.9	11.1
Ca	0.2	6.3	K_2_O	6.3	0.6
Na	1.0	2.3	Na_2_O	1.6	3.4
Mn	<0.1	0.1	CuO	1.7	1.6
Mg	<0.1	2.2	ZnO	1.3	1.3
Zn	<0.1	<0.1	NiO	1.0	1.9
Cr	<0. 1	<0.1	MnO	0.1	0.2
Cu	<0. 1	<0.1	MgO		4.1
Co	<0.1	<0.1	TiO_2_		2.3
Cd	<0.1	<0.1			
Ni	<0.1	<0.1			
Pb	<0.1	<0.1			
As	<0.1	<0.1			

**Table 2:** Parameters of the pseudo-first-order and pseudo-second-order kinetic models for As(V) adsorption by Ch–Rs and Ch–Pu blends.

Parameter	Ch–Rs				Ch–Pu			
	Pseudo-First-Order		Pseudo-Second-Order		Pseudo-First-Order		Pseudo-Second-Order	
*C*_0_ (mg/L)	0.25	2.0	0.25	2.0	0.25	2.0	0.25	2.0
*q_e_*_,*exp*_(mg/g)	0.032	0.234	0.032	0.234	0.028	0.224	0.028	0.224
*q_e_*_,*cal*_(mg/g)	0.030	0.234	0.031	0.235	0.028	0.224	0.028	0.225
*k*_1_ (min^−1^)	0.628	0.582	-	-	0.536	0.574	-	-
*k*_2_(g/(mg min))	-	-	126.88	12.72	-	-	85.36	13.34
*V*_0_(mg/(g min))	-	-	0.130	0.697	-	-	0.067	0.669
*R*^2^	0.80706	0.77013	0.94773	0.9303	0.8837	0.88446	0.98627	0.96758
*χ*^2^	4.93 × 10^−8^	5.76 × 10^−6^	1.34 × 10^−8^	1.75 × 10^−6^	5.99 × 10^−8^	2.55 × 10^−6^	7.07 × 10^−9^	7.17 × 10^−7^

**Table 3:** Isotherm parameters of the adsorption of As (V) on Ch–Rs and Ch–Pu blends.

Isotherm	Parameters	Ch–Rs	Ch–Pu
Langmuir	*q_max_* (mg/g)	0.722	0.710
	*B* (L/mg)	1.551	1.712
	*R_L_*	0.06–0.86	0.06–0.85
	*R*^2^	0.997	0.999
	*χ*^2^	8.34 × 10^−3^	8.24 × 10^−3^
Freundlich	*K_F_* ((mg^1−1/*n*^ L^1/*n*^)/g)	0.361	0.363
	*n*	2.318	2.374
	*R*^2^	0.953	0.938
	*χ*^2^	2.618	2.722

**Table 4:** Comparison of maximum adsorption capacity (*Q_max_*, mg/g) of various low cost adsorbents for As (V) removal.

Adsorbent	Initial pH	*Q_max_* (mg/g)	References
Feldspar	3	0.24	[63]
Manganese oxide coated zeolite	7	0.15	[64]
Magnetic iron oxide nanoparticles coated on sand	7	0.29	[65]
Iron-oxide coated sands	7	0.021	[66]
Fe(III)–Sn(IV) mixed oxide-coated sand	7	0.23	[67]
Kaolinite		0.86	[68]
Laterite soil	5.7	0.04	[69]
Rice polish	4	0.15	[70]
Modified zeolite Y	6	1.34	[71]
Fish scale	4	0.027	[72]
Bone char	10	1.43	[73]
Red mud	3.5	0.52	[74]
Red mud	2.3	0.51	[75]
Natural Muscovite	6	0.79	[76]
Iron oxide coated sand	7	0.099	[77]
Ch–Rs	7	0.72	This study
Ch–Pu	7	0.71	This study
